# A direct negative feedback loop of miR-4721/FOXA1/Nanog promotes nasopharyngeal cell stem cell enrichment and metastasis

**DOI:** 10.1186/s12967-021-03059-y

**Published:** 2021-09-09

**Authors:** Mengyang Zhao, Zibo Tang, Yijun Wang, Jiaojiao Ding, Ying Guo, Tianhui Gao

**Affiliations:** 1 Department of Oncology, Henan Provincial People’s Hospital, People’s Hospital of Zhengzhou University, Zhengzhou, 450003 Henan China; 2 Cancer Center, Traditional Chinese Medicine-Integrated Hospital of Southern Medical University, Guangzhou, 510000 China

**Keywords:** miR-4721, FOXA1, Nanog, NPC, Invasion, Migration, CSCs

## Abstract

**Objective:**

The recurrence and metastasis of nasopharyngeal cancer (NPC) may be mainly attributed to the persistence of cancer stem cells (CSCs); however, the linkage mechanism has yet to be fully elucidated.

**Methods:**

The levels of miR-4721, FOXA1, and Nanog expression in NPC were detected by in situ hybridization and immunohistochemistry. In vivo and in vitro metastasis assays confirmed miR-4721 promotes cell migration and invasion. Tumor spheroid formation assay, side population (SP) assay, and ALDEFLUOR assay verified miR-4721 regulates cancer stem cell-like properties. Luciferase reporter assay showed that miR-4721 directly regulates FOXA1 and FOXA1 effects the promoter activity of miR-4721 and Nanog. Chromatin immunoprecipitation (ChIP) analysis and electrophoresis mobility shift assay (EMSA) revealed that FOXA1 combined the promoter region of human miR-4721 and Nanog and the possible mechanism was also analyzed.

**Results:**

In this study, a new mechanism of NPC tumorigenesis related to miR-4721 was verified. We found that miR-4721, FOXA1 and Nanog control their expressions through a negative feedback loop and then activate the downstream regulator of stem cell signaling to promote the enrichment and metastasis of NPC stem cells.

**Conclusion:**

These findings elucidate that the feedback loop of miR-4721/FOXA1/Nanog can regulate stemness and metastasis in NPC and may provide an experimental theoretical basis for metastasis and treatment resistance in NPC.

**Supplementary Information:**

The online version contains supplementary material available at 10.1186/s12967-021-03059-y.

## Introduction

Cancer stem cells (CSCs) comprise a small part of tumor tissues capable of self-renewal, immortal proliferation, and differentiation. They are the root cause of tumor recurrence, metastasis, and treatment tolerance [1, 2]. Cancer stem cells seem to be involved in all stages of tumor formation, development, and spread [3]. The transformation of epithelial cells to mesenchymal cells (EMT) can promote tumor cell infiltration and tumor metastasis. EMT and the stem cell state of tumor cells are intertwined [4].

Nasopharyngeal carcinoma (NPC) is a malignant tumor that originates from epithelial cells located in the nasopharynx [5]. The incidence of nasopharyngeal cancer has obvious regional and ethnic differences. Yellow people are most susceptible, and Caucasians are very rare. The incidence of nasopharyngeal cancer is the highest in East Asia and Africa, but rare in other regions. The occurrence of NPC is suggested to be potentially related to the Epstein–Barr virus (EBV) [6], environmental factors, genetic factors, eating habits, etc. Current research indicates that the abnormal expression of miRNAs is widely involved in the pathological process of NPC [7]. At present, miRNAs closely related to the development of NPC include miR-29c, miR-200a, miR-15b, miR-20b, miR-204, miR-3188 [8], miR-374a [9], and so on. An in-depth study on the relationship between miRNA and the pathogenesis of NPC can provide an updated perspective on the occurrence and development of NPC.

A growing volume of evidence suggests that miRNA can affect the stem cell status of tumor cells. Therefore, miRNA can also link the stem cell status of tumor cells to their metastatic process via EMT regulation because the genetic development of EMT is related to both the stem cell status and tumor metastasis [10]. MiRNA regulation of tumor stem cell differentiation and self-renewal ability has been confirmed in prostate cancer stem cells [11], breast cancer stem cells, leukemia stem cells, and pancreatic cancer stem cells. The role of miRNA in mediating the transfer of tumor stem cells has attracted research interest. On the basis of a large volume of research, we speculate that the same miRNA may not only be involved in maintaining the phenotype of cancer stem cells but in tumor cell infiltration and metastasis as well. Our previous research indicated that miR-133a-3p and miR-200b involved in the process of stemness and metastasis of NPC [12]. We also found EBV-miR-BART22 promoted NPC stemness and metastasis [13], and microarray chip analysis showed miR-4721 was upregulated in miR-BART22-overexpressed NPC cells. Furthermore, we confirmed that miR-4721 overexpressing was able to promote NPC cell growth and was significantly involved in NPC progression [14]. However, whether miR-4721 is associated with stemness and metastasis in NPC is not clear.

In this study, the relationship between miR-4721, FOXA1 and Nanog in NPC was confirmed, and we found that miR-4721, FOXA1, and Nanog regulated their expressions through a negative feedback loop and then activate the downstream regulator of stem cell signaling to promote the stemness and metastasis of NPC cells.

## Materials and methods

### Cell culture and tissue specimens

HONE1 and SUNE1 cells were cultured in RPMI-1640 (Invitrogen) containing 10% fetal calf serum (FCS; Hyclone, Invitrogen). HONE1 and SUNE1 cells were cultured at 37 °C in a 5% CO_2_ incubator. Tissue microarrays including 132 NPC samples were purchased from Outdo BiotechCo., Ltd, Shanghai, China.

### In situ hybridization

The expression of miR-4721 in 132 cases of nasopharyngeal carcinoma tissues was examined by in situ hybridization in Bioscience (Guangzhou, China). The positive staining was detected by a diaminobenzidine (DAB) substrate kit (AxyBio, Guangzhou, China).

### Transient transfection with FOXA1 or miR-4721 mimics/inhibitor

RiboBio Inc. (Guangzhou, China) and Biosense Technologies (Guangzhou, China) designed and synthesized the mimics and inhibitor of miR-4721 and the plasmid of FOXA1. The sequence of miR-4721 and its control are listed in Additional file 1: Table S1. The specific experimental steps are the same as before [14].

### Western blotting

Follow the steps (https://www.westernblotprotocol.com) for western blotting. The following antibodie were used: rabbit anti-FOXA1 (1:1000; CST, USA), rabbit anti-Nanog (1:1000; CST, USA), rabbit anti-Snail (1:1000; CST, USA), rabbit anti-ZEB2 (1:1000; CST, USA), rabbit anti-E-Cadherin (1:1000; CST, USA), mouse anti-β-actin (1:5000; Beyotime AA128, China). The antibodies are listed in Supplementary Table 3.

### Tumor spheroid formation assay

NPC cells (1000 cells/ml) were cultured in serum-free Ham’sF-12 medium (Gibco) supplemented with 20 ng/ml epidermal growth factor (EGF, Invitrogen, Grand Island, NY, USA), B27 (1:50; Gibco), and 20 ng/ml fibroblast growth factor (FGF, Invitrogen). After 72 h of culture, tumorspherers were counted under a microscope.

### Side population assay

1 × 10^6^ nasopharyngeal carcinoma cells/ml are suspended in DMEM/2% FBS. Then mixed into a single cell suspension, alone or in combination with verapamil (50 mmol/ml; Sigma-Aldrich), and then incubated with Hoechst 33342 dye (5 μg/ml; Sigma-Aldrich) at 37 °C for 90 min. After incubation, the cells were stained with propidium iodide (1 μg/ml; Sigma Aldrich). The FACS Aria flow cytometer (BD Biosciences) was used for flow cytometry analysis.

### Establishment of subcutaneous xenograft mouse model

To determine the tumor formation ability, a mouse model of subcutaneous xenograft tumors was established. 1 × 10^6^, 5 × 10^5^, 1 × 10^5^, and 5 × 10^4^ NPC cells were respectively injected into mice (N = 6 per group). According to a previous description (http://bioinf.wehi.edu.au/software/elda/), Extreme limiting dilution analysis were performed to assess tumor-initiating frequencies.

### ALDEFLUOR

The ALDEFLUOR assay kit (Stem Cell Technologies) was used to detect the activity of ALDH. Single cells isolated from cell lines or tumor samples were mixed in ALDEFLUOR assay buffer (1.5 µM) containing the ALDH substrate BODIPY amino acetaldehyde (BAAA) and incubated for 1 h at 37 °C. Diethylamino benzaldehyde (DEAB), was used as a negative control (tenfold molar excess). BD FACS Diva software V6.1.3 (BD Biosciences) or Flow Jo software (Tree Star) were used to analyze flow cytometry data.

### Immunofluorescent staining

According to the previous description, Immunofluorescent staining assays were performed [13]. Store the slides in a dark box at 4 °C and observe under a fluorescence microscope.

### Immunohistochemical staining

IHC is used to assess the expression level of FOXA1 and Nanog in samples of NPC and its control tissues, which has been previously described [14]. Two pathologists who were blind to the clinical parameters examined the stained tissue sections and scored them separately. The staining scoring criteria are also described [14]. In order to statistically analyze the expression of FOXA1 and Nanog in NPC tissues in non-cancerous tissues, the staining scores 0–4 and 5–6 are regarded as low expression and high expression, respectively.

### Luciferase reporter assay

Luciferase reporter assay was performed to show that miR-4721 directly regulates FOXA1. The WT 3ʹUTR or mutant 3ʹUTR was cloned into psiCHECK-2 vectors. The WT or mutant 3ʹUTR vector was co-transfected with miR-4721 mimics/inhibitors or a non-specific control into NPC cells. To investigate whether FOXA1 effects the promoter activity of miR-4721 and Nanog, we constructed the Nanog promoter-luciferase reporter plasmid pGL3-Nanog and the miR-4721 promoter-luciferase reporter plasmid pGL3-miR-4721. Luciferase activity was measured 48 h after transfection using the Dual-Luciferase Reporter Assay System (Promega, Madison, WI, USA). The primers sequences used in the luciferase activity reporter assay are listed in Additional file 1: Table S2.

### CHIP assay

According to the previous description [8], we used was using a ChIP assay kit (Millipore) to perform ChIP assay according to the manufacturer’s instructions. DNA–protein complexes were immunoprecipitated from NPC cells with antibodies anti-FOXA1 or IgG (Cell Signaling Technology, Danvers, MA, USA). The enrichment of the miR-4721 and Nanog promoter region were measured by qRT-PCR analysis and PCR analysis. PCR Primers are listed in Additional file 1: Table S2.

### EMSA analysis

An EMSA kit (Roche Diagnostics, Basel, Switzerland) was used to detect the binding site of FOXA1 to the promoters of miR-4721 and Nanog according to the manufacturer’s instructions. According to the previous description [8], Biosense Bioscience (Guangzhou, China) completed EMSA analysis. Summary of the sequences of the probes used in the EMSA assay were seen in Additional file 1: Table S4.

### Statistical analysis

Data analysis were completed by IBM SPSS v20.0 (IBM Corporation, Armonk, NY, USA) and GraphPad Prismv5.0 (GraphPad Software, Inc., La Jolla, CA, USA) software. Chi-square test analysis of the relationship between FOXA1 expression level and clinicopathological characteristics. Two independent group was compared by wo-tailed Student’s t-test. The differences between groups for all in vitro analyses were determined by One-way ANOVA. Differences were considered significant if *P* < 0.05.

## Results

### MiR-4721 regulates cancer stem cell-like properties in NPC

**Fig. 1:**
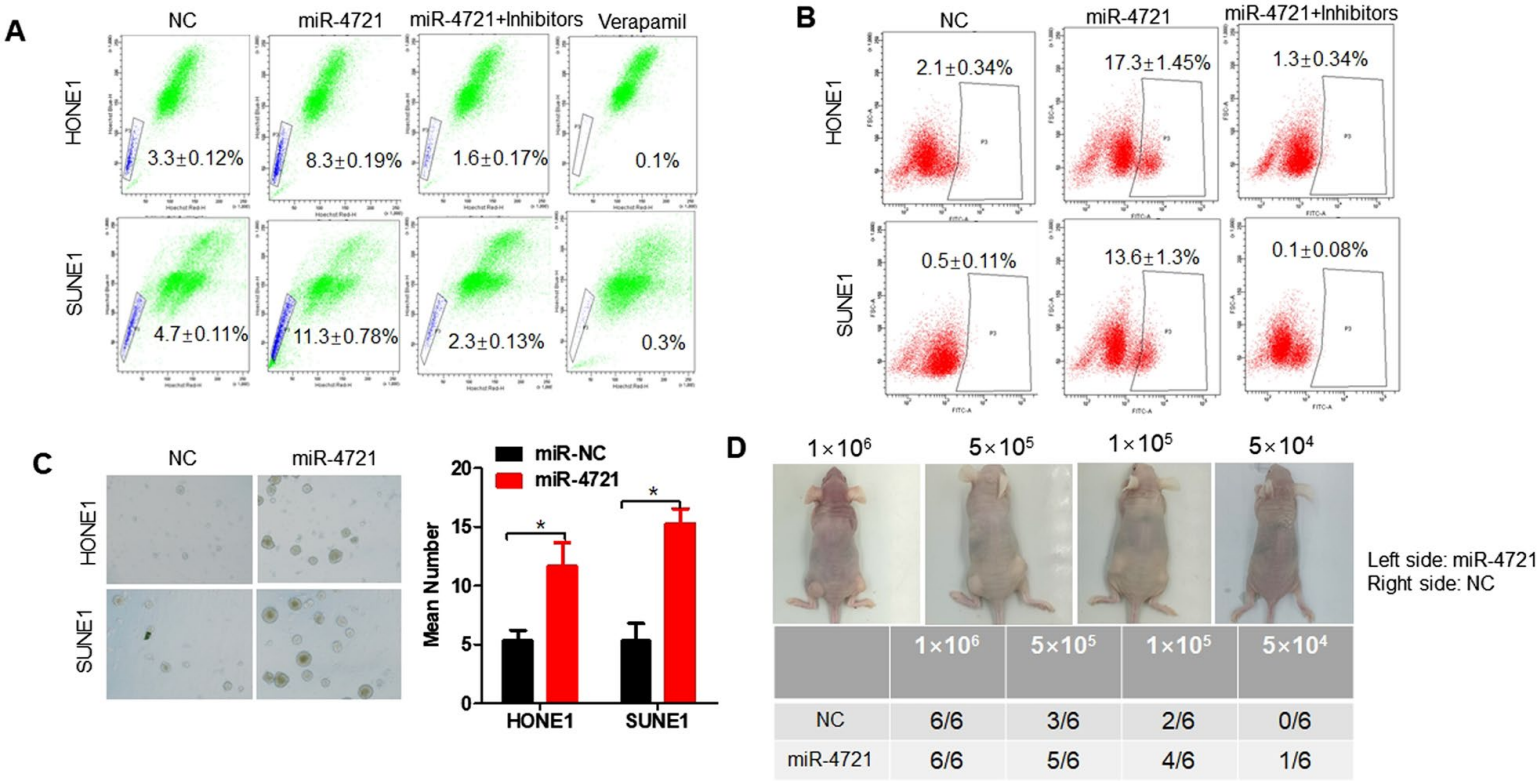
miR-4721 expression promotes stem cell enrichment in HONE1 and SUNE1 cells. **A** Analysis of SP cells from miR-4721-overexpressing or miR-4721-inhibited, and NC cells by flow cytometry. **B** ALDEFLUOR assay of Aldehyde dehydrogenase-positive cells of miR-4721-overexpressing or miR-4721-inhibited, and NC cells. **C** Representative images of the tumor sphere seeded at a density of 1000 cells/well for 7 days. **D** The nude mice were injected with 10^6^, 5 × 10^5^, 10^4^, or 5 × 10^4^ miR-4721-overexpressing cells or control cells on the left and right sides, respectively (n = 6). *P < 0.05

Side population (SP) cells exhibit possess NPC CSC properties [15]. Research confirmed that NPC tumor sphere cells cultured in serum-free cultures showed a significant increase in the proportion of SP cells relative to that of the parent cells (Fig. 1A), and adding inhibitors to NPC cells overexpressing miR-4721 reversed this effect. Previous research confirmed aldehyde dehydrogenase 1 (ALDH1) was a functional marker for identifying cancer stem cells in human nasopharyngeal carcinoma [16]. We found that ALDH1 + labeled cells were significantly increased among overexpressing miR-4721 HONE1 and SUNE1 cells compared with those control cells, and these results could be reversed when adding inhibitors to NPC cells overexpressing miR-4721(Fig. 1B). Further, the sphere formation ability was enhanced in overexpressing miR-4721 HONE1 and SUNE1 cells (Fig. 1C). In the xenograft model, the estimated tumor initiation frequency was 5 × 10^4^ when inoculated with HONE1-miR-4721 cells, whereas when inoculated with control cells, the tumor initiation was 1 × 10^5^ (Fig. 1D). All the results show that miR-4721 regulated the CSC-like properties in NPC.

### MiR-4721 promotes cell migration and invasion in vitro and in vivo

**Fig. 2:**
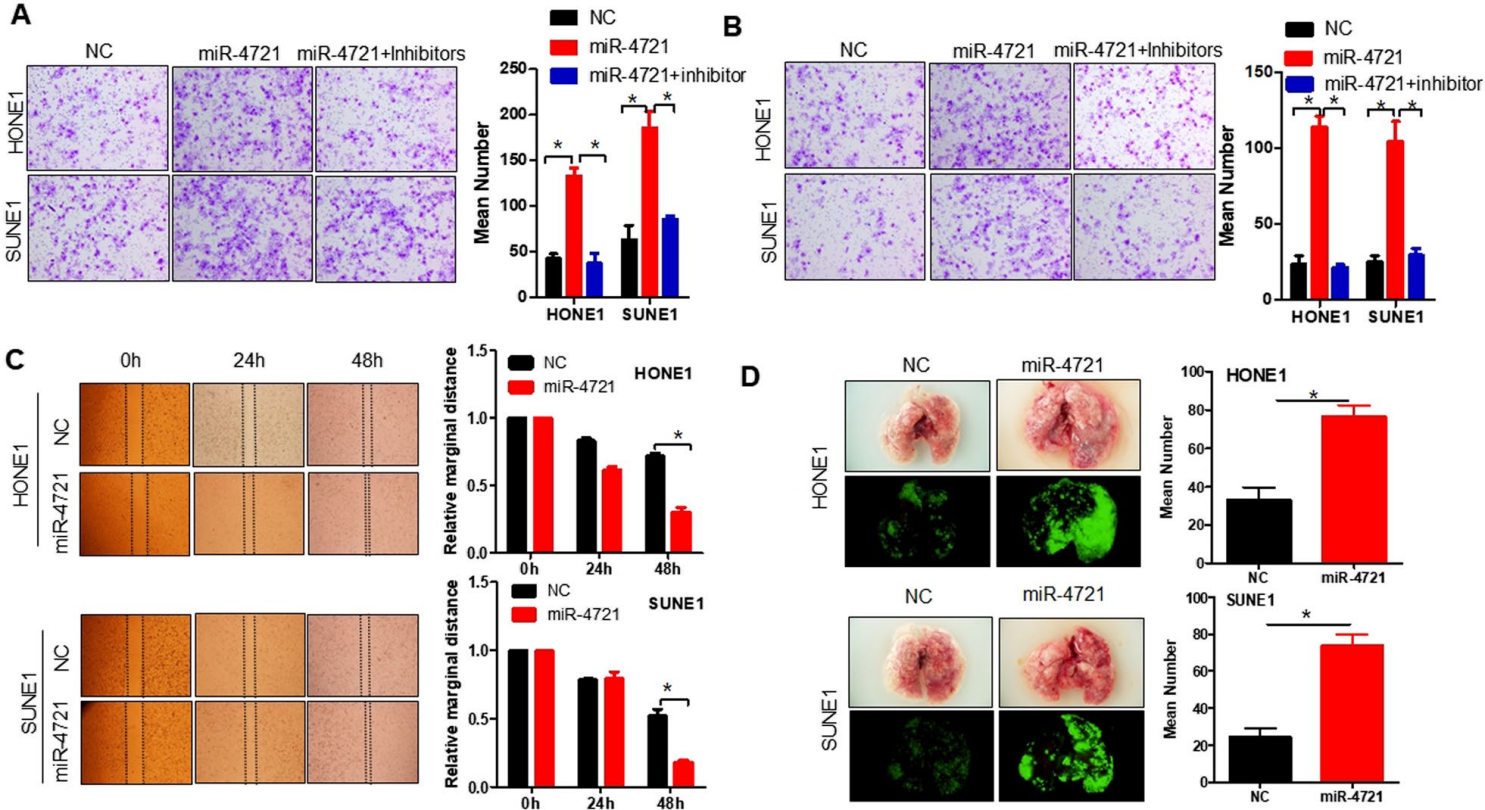
miR-4721 promotes HONE1 and SUNE1 migration and invasion in vivo and in vitro. Transwell assay (**A**) and Boyden Chamber assays (**B**) and wound healing assays (**C**) were performed to certify the ability of miR-4721 on NPC cell migration and invasion. **D** Representative bioluminescence imaging of the lungs collected from mice that received transplants of indicated cells and euthanized on Day 30. *P < 0.05

Migration and invasion assays were conducted to evaluate the effects of miR-4721 on cancer cell migration and invasive ability, the results showed that miR-4721 overexpression markedly enhanced the motility and invasiveness of HONE1 and SUNE1 cells relative to those of their control cells (*P* < 0.05; Fig. 2A, B). However, the addition of miR-4721 inhibitor to NPC cells overexpressing miR-4721 could in turn reduce the invasion and migration ability of miR-4721-overexpressing NPC cells (*P* < 0.05). In addition, in a wound-healing assay, overexpression of miR-4721 significantly accelerates the migration of NPC cells at the edge of the scratch wound (Fig. 2C). Quantitative analysis at 48 h confirmed that wound closure was significantly reduced in miR-4721 overexpressing cells compared to control cells transfected with empty vector (*P* < 0.05; Fig. 2C). Therefore, miR-4721 markedly promoted the migratory and invasive abilities of NPC cells in vitro.

To assess the effect of miR-4721 on NPC metastasis in vivo, we injected miR-4721 overexpressed NPC cells and their control cells into the spleen of nude mice. Fluorescence imaging indicated that intrapulmonary metastatic nodules in large quantities were generated in the mice injected with HONE1-miR-4721 and SUNE1-miR-4721 cells, whereas few small clusters were observed in HONE1 and SUNE1 control cells (*P* < 0.05; Fig. 2D). Similar to in vitro results, miR-4721 promoted the metastasis of NPC cells.

### FOXA1 is a novel target for miR-4721

**Fig. 3:**
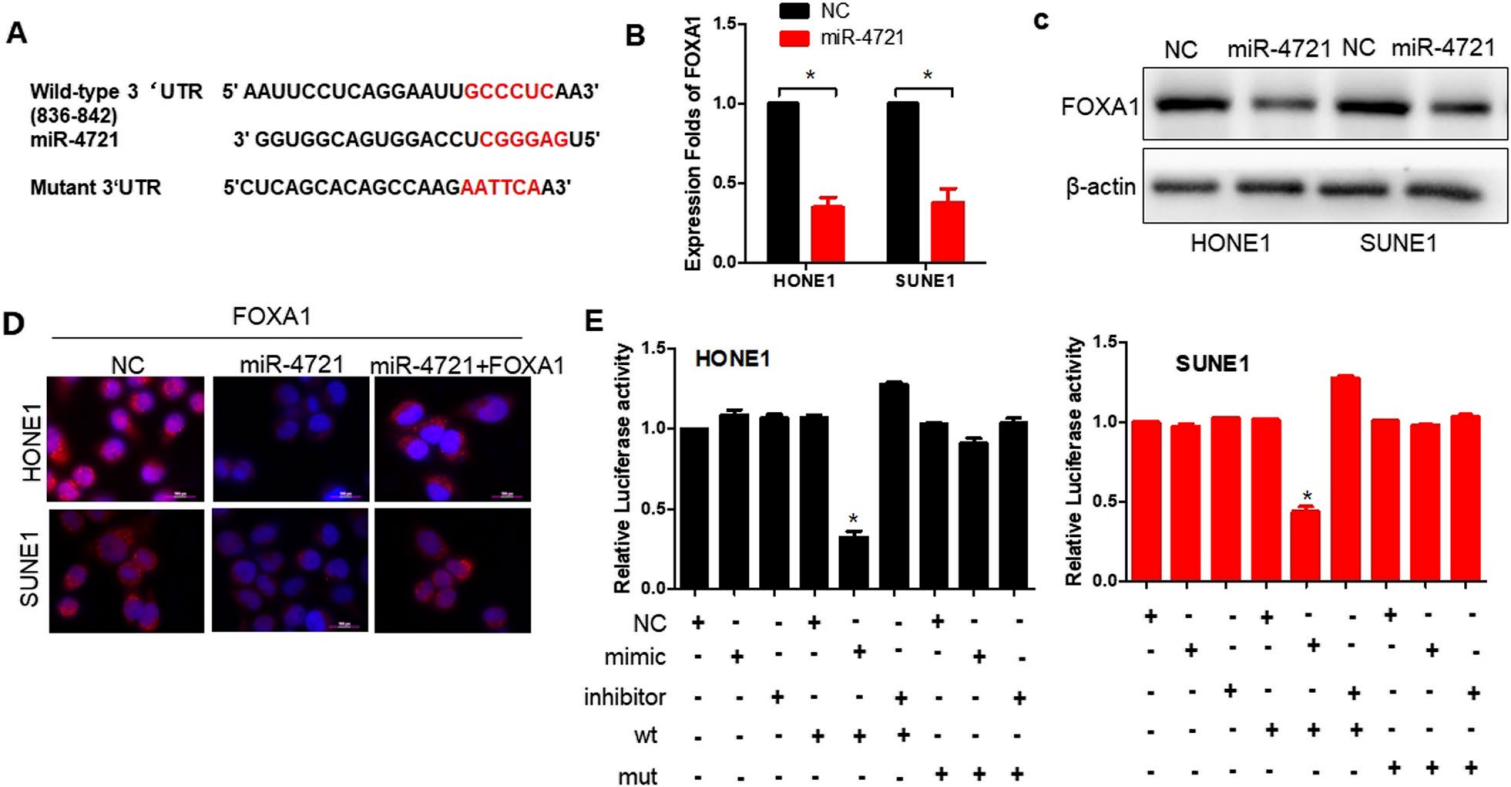
miR-4721 directly regulates FOXA1. **A** The putative binding sequences of miR-4721 in the 3'UTR of FOXA1. Mutation in the complementary site that binds to the seed region of miR-4721. **B** QPCR is used to detect the expression of FOXA1 in miR-4721-overexpressing NPC cells, normalized to U6. **C** Western blot analysis of FOXA1 in miR-4721-overexpressing NPC cells, with β-actin as a loading control. **D** Immunofluorescence staining of FOXA1 after transfection with NC, ectopic FOXA1, and miR-4721 as indicated. Scale bar, 100 px. (E) miR-4721 directly targeting the FOXA1 3ʹUTR, as determined by luciferase reporter assay. *P < 0.05

FOXA1 was a direct target of miR-4721, as predicted using the TargetScan and RNAhybrid algorithms (Fig. 3A). Overexpression of miR-4721 downregulated the mRNA and protein levels of FOXA1 in HONE1 and SUNE1 cells (Fig. 3B, C). Immunofluorescence was used to confirm the reduced expression of FOXA1 in MiR-4721-overexpressing NPC cells (Fig. 3D). Ectopic expression of FOXA1 with plasmid-FOXA1 partly rescued the miR-4721-mediated reduction in FOXA1. Similarly, cotransfection of miR-4721 mimics significantly decreased FOXA1 luciferase reporter activity (Fig. 3E, lane 5; *P* < 0.05), whereas the opposite effect were shown in miR-4721 inhibitors (Fig. 3E, lanes 3 and 6). These results collectively suggest that miR-4721 exerted its effects on NPC by direct FOXA1 suppression.

### FOXA1 binds the promoter region of human miR-4721

**Fig. 4:**
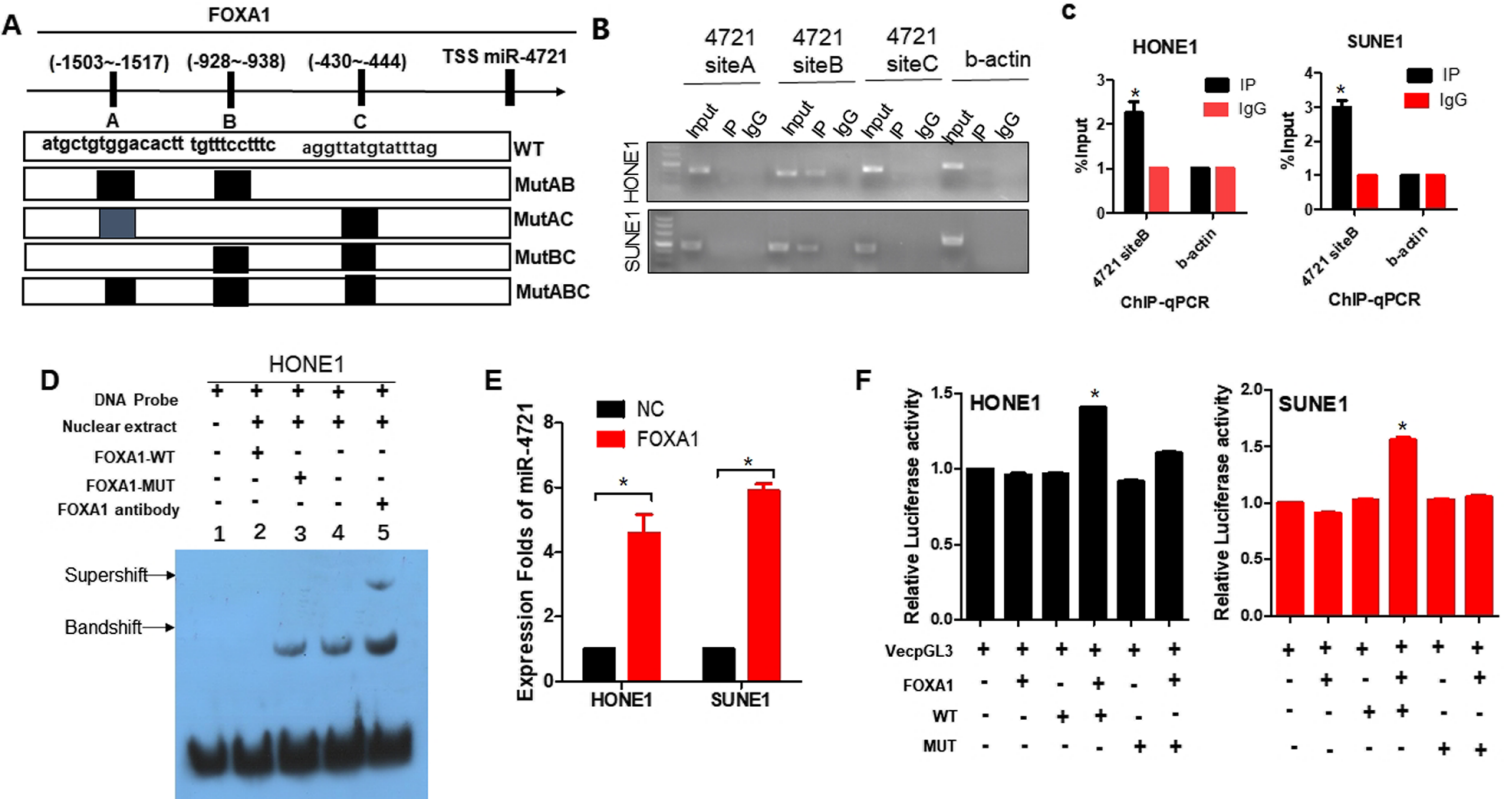
Confirm FOXA1 binds to the miR-4721 promoter region. **A** Schematic diagram of the miR-4721 promoter region with putative FOXA1 TFBS (**A**, **B**, and **C**) and wild-type (WT) and TFBS mutants (MutAB, MutAC, MutBC, and MutABC). **B** CHIP experiment shows 3 binding sites of FOXA1 in miR-4721 promoter region. **C** Amplification of miR-4721-binding sites after Ch-IP using an antibody against FOXA1. An IgG antibody was used as the negative control. **D** EMSA assay of FOXA1 binding to miR-4721 promoter in NPC cells. **E** The expression level of miR-4721 in NPC cells overexpressing FOXA1 was detected by qPCR. **F** Relative luciferase activity of the indicated promoter vectors HONE1 and SUNE1 cells transfected with FOXA1 plasmids. *P < 0.05

We further investigated the potential relationship between miR-4721 and FOXA1 using bioinformatics. The 2 kb region upstream of the miR-4721 transcription start site (TSS) was analyzed by TESS, UCSC, and TFSEARCH, we found that the promoter region of miR-4721 has 3 transcription factor–binding sites (TFBS) for FOXA1. The 3 TFBSs were designated as A, B, and C (Fig. 4A). Chromatin immunoprecipitation (ChIP) analysis revealed that FOXA1 was most significantly bound to the TFBS B of the miR-4721 promoter (Fig. 4B, C), confirming the direct interaction between FOXA1 and miR-4721.

Further, EMSA experiments were performed to determine whether HONE1 nuclear extracts could bind to the predicted site B. As shown in Fig. 4D, the mutant competition group (lane 3), experimental group (lane 4), and super shift group (lane5) had binding bands, whereas all results of the negative control group (lane 1) and wild-type competition group (lane 2) were negative, indicating that the transcription factor FOXA1 was bound to the target gene miR-4721 promoter. Furthermore, qPCR confirmed that the expression level of miR-4721 was significantly increased after overexpression of FOXA1 (Fig. 4E). The wild-type miR-4721 promoter luciferase activity was further elevated when FOXA1 was upregulated in the HONE1 and SUNE1 cell lines (*P* < 0.05)**.** However, when B was mutated, overexpression of FOXA1 did not further increase the activity of luciferase (Fig. 4F). These data indicate that FOXA1 binds to the specific promoter TFBS of miR-4721 and accelerates transcription. These results showed a negative feedback loop to control the expression of FOXA1 and miR-4721, thereby regulating the stemness and metastasis of NPC.

### FOXA1 binds the promoter region of Nanog and regulates CSC-related gene

**Fig. 5:**
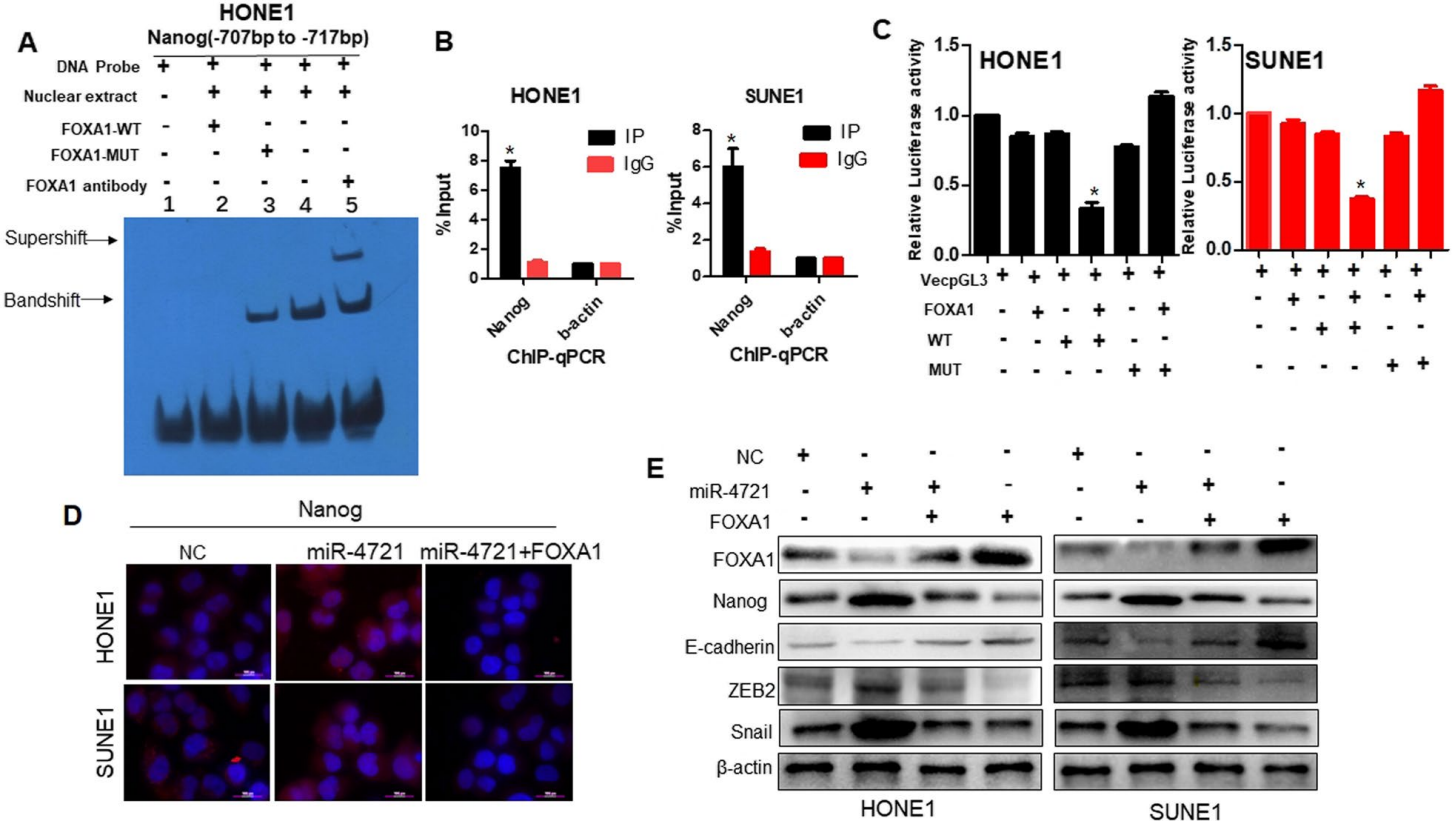
miR-4721 regulates FOXA1-modulated CSC-associated genes and EMT markers. **A** EMSA assay of FOXA1 binding to Nanog promoter in NPC cells. **B** CHIP experiment shows FOXA1 binds miR-4721 promoter region. **C** Relative luciferase activity of the indicated promoter vectors HONE1 and SUNE1 cells transfected with FOXA1 plasmids. **D** Immunofluorescence staining of Nanog after transfection with NC, ectopic FOXA1, and miR-4721 as indicated. Scale bar, 100 px. **B** miR-4721 modulates CSC-associated genes and EMT markers via the FOXA1/NANOG pathway in HONE1 and SUNE1. E-cadherin, ZEB2, and Snail with NC, ectopic FOXA1, and/or miR-4721 as indicated in NPC cells by Western blot analysis

FOXA1 cooperates or interacts with certain genes to control the differentiation of embryonic stem cells. Chen et al. found that FoxA1 and Grg3 endogenously bound to the Nanog promoter near -2 kb in vitro [17]. This finding prompted our interest in whether miR-4721 participated in the promotion of the metastasis and stem cell enrichment of NPC cells via the FOXA1/NANOG pathway. The -2 kb region upstream of the Nanog gene was scanned for potential binding sites for FOXA1 using Mac Vector software. The scan revealed that there were multiple tandem potential binding sites for FOXA1 in the region from − 707 to − 717 bp. We then used EMSA and ChIP assays to examine whether FOXA1 bind to this region. EMSA experiments were performed to determine whether HONE1 nuclear extracts could bind to the predicted site. As shown in Fig. 5A, the mutant competition group (lane 3), experimental group (lane 4), and super shift group (lane5) had binding bands, whereas all results of the negative control group (lane 1) and wild-type competition group (lane 2) were negative, indicating that the transcription factor FOXA1 was bound to Nanog promoter. ChIP analysis further revealed that FOXA1 was most significantly bound to the promoter of Nanog (*P* < 0.05; Fig. 5B), confirming the direct interaction between FOXA1 and Nanog. Further, the Nanog − 2.3 kb promoter was cloned, and it was confirmed by dual-luciferase reporter assay that FOXA1 inhibited Nanog promoter activity (*P* < 0.05; Fig. 5C). These data indicate that FOXA1 binds to the specific promoter TFBS of Nanog and inhibits transcription.

Furthermore, we measured the levels of the FOXA1- and CSC-related gene NANOG in miR-4721, miR-4721-NC, miR-4721 + FOXA1, and FOXA1 NPC cells by Western blot analysis and immunofluorescence assay. Meanwhile, the levels of E-cadherin and ZEB2, and snail were also detected by Western blot analysis. As shown in Fig. 5D, Immunofluorescence was used to confirm the increased expression of Nanog in MiR-4721-overexpressing NPC cells (Fig. 5D). Ectopic expression of FOXA1 with plasmid-FOXA1 partly rescued the miR-4721-mediated and increase in NANOG. Western blot analysis indicated that the upregulation of miR-4721 inhibited the expression of FOXA1 and E-cadherin a relative to that of the control cells; such inhibition of FOXA1 and E-cadherin by miR-4721 was restored by FOXA1 (Fig. 5E). Meanwhile, the upregulation of miR-4721 increased the expression of ZEB2, and Snail in HONE1 and SUNE1 cells. Suppressing miR-4721 by overexpressing FOXA1 reversed the effects mediated by ectopic miR-4721 (Fig. 5E). Moreover, FOXA1 upregulation inhibited the expression of NANOG, ZEB2, and Snail, but increased the expression of E-cadherin. These combined data suggest that in HONE1 and SUNE1 cells, miR-4721, FOXA1 and Nanog control their expressions through a negative feedback loop and then activate the downstream regulator of stem cell signaling.

### Correlation of miR-4721, FOXA1, and Nanog in NPC specimens

**Fig. 6:**
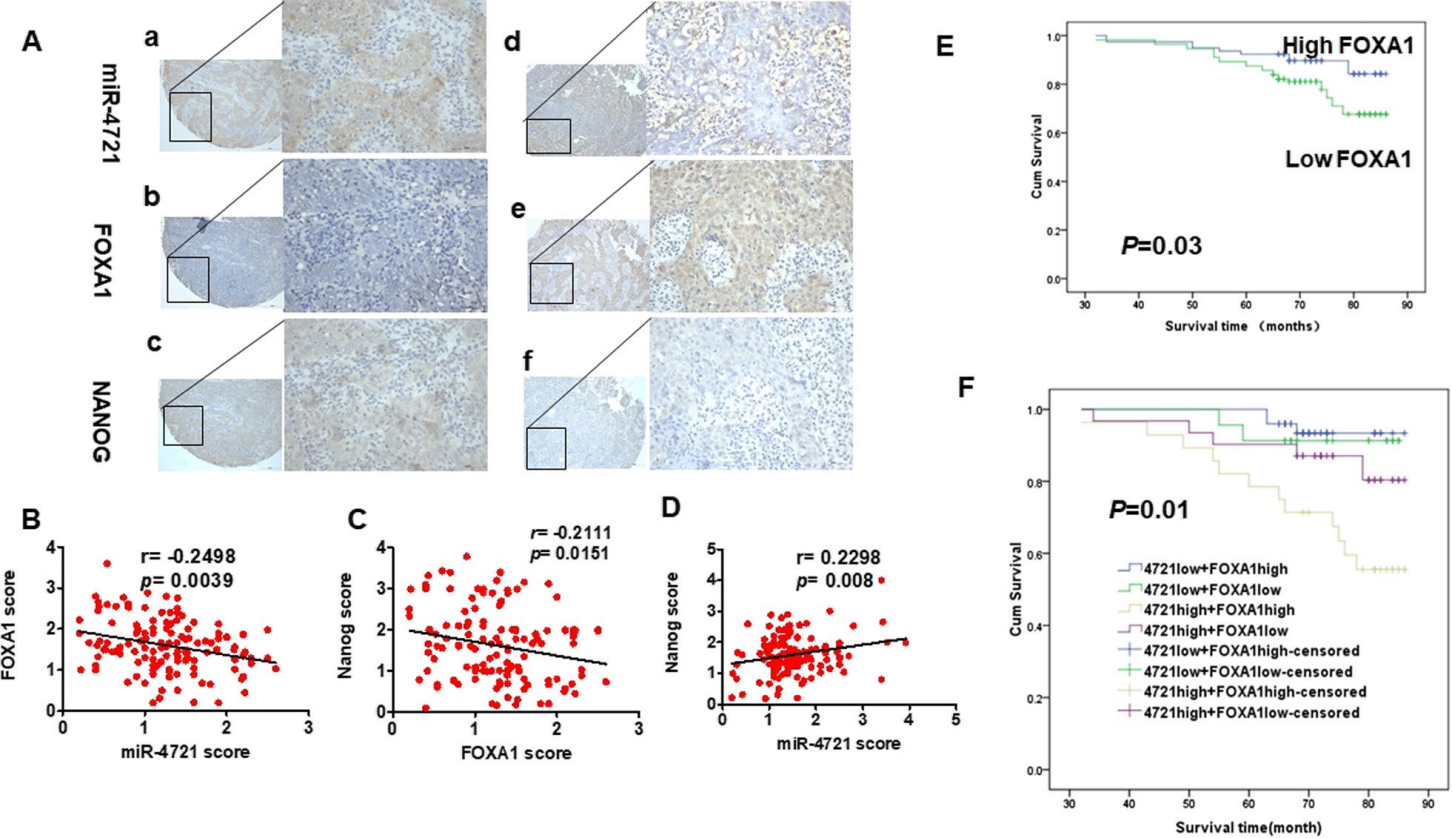
Relationship between miR-4721, FOXA1 and Nanog. **A** Expression levels of miR-4721, FOXA1 and Nanog in NPC specimens, as detected by in situ hybridization and immunohistochemistry. a: strong expression of miR-4721; b: low staining of FOXA1; c: strong expression of Nanog; d: low staining of miR-4721; e: strong expression of miR-FOXA1; f: low staining of Nanog; The scale bars are 200 and 50 μm respectively. **B** Two-tailed Spearman’s correlation analysis was performed to calculate the correlations between FOXA1 and miR-4721 expression levels. *P* = 0.0039; *r* = − 0.2498. **C** Two-tailed Spearman’s correlation analysis was performed to calculate the correlations between Nanog and FOXA1 expression levels. *P* = 0.0151; *r* = − 0.2111. **D** Two-tailed Spearman’s correlation analysis was performed to calculate the correlations between Nanog and miR-4721 expression levels. *P* = 0.0008; *r* = 0.2298. **E** According to the FOXA1 protein expression, Kaplan–Meier survival analysis was performed on the overall survival of 132 NPC patients. **F** Patients with low miR-4721 expression and high FOXA1 expression survive longer in NPC specimens. **P < 0.01

In our previous research, we found miR-4721 is obviously related to clinical staging of NPC patients; moreover, NPC patients with low miR-4721 expression had longer survival than those with high miR-4721 expression [14]. Furthermore, we determined whether miR-4721 expression was associated with FOXA1 and Nanog expression in human NPC tissues (Fig. 6A). Spearman's correlation analysis showed that miR-4721 is negatively correlated with FOXA1 (Fig. 6B, r = − 0.2498, *P* = 0.0039) and FOXA1 is negatively correlated with Nanog levels (Fig. 6C, r = − 0.2111, *P* = 0.0151). Meanwhile, we also found a positive correlation between miR-4721 and Nanog (Fig. 6D, r = 0.2298, *P* = 0.008). In addition, we analyzed the relationship between clinicopathological characteristics and FOXA1 expression in NPC patients. FOXA1 is obviously related to lymph node metastasis (N classification; N0–N1 vs. N2–N3; *P* = 0.042) (Table 1).

**Table 1:** Correlation between the clinicopathologic characteristics and expression of FOXA1 in NPC

Characteristics	n	FOXA1 expression		*P* value
		High	Low	
Age, years				
< 50	74	29	45	0.592
≥ 50	58	20	38	
Gender				
Male	101	40	61	0.396
Female	31	9	22	
Clinical stage				
I–II	72	26	46	0.0857
III–IV	60	23	37	
N stage				
N0–N1	36	8	28	**0.042**
N2–N3	96	41	55	
M stage				
M0	2	0	2	0.530
M1	130	49	81	

*NPC* nasopharyngeal carcinoma, *TNM* tumor, node, metastasis

*P* values by t test

Statistically significant values are in bold

As listed in Table 2, Multivariate analysis showed that the level of FOXA1 was significantly associated with overall survival. Patients with low levels of FOXA1 had longer survival than those with high levels of FOXA1 (Fig. 6E, P = 0.03). Furthermore, we examined the prognostic value of miR-4721 for different subgroups of NPC samples. In subgroup analysis, patients with low miR-4721 expression had longer survival than that of patients with high miR-4721 expression, regardless of their FOXA1 expression (Fig. 6F, P = 0.01).

**Table 2:** Univariate and multivariate analysis of the influence of various clinicopathological characteristics on the prognosis of patients

Characteristics	Univariate analysis			Multivariate analysis		
	*P*	HR	*95%CI*	*P*	HR	*95%CI*
Age						
< 50 vs ≥ 50 years	0.153	0.538	0.23–1.258			
Gender						
Male vs female	0.529	0.670	0.192–2.333			
Clinical stage						
I–II vs III–IV	0.642	1.220	0.528–2.815			
N stage						
N0–N1 vs N2–N3	0.039	8.277	1.113–61.54	0.090	5.804	0.762–44.233
M stage						
M0 vs M1	0.053	7.460	0.975–57.052			
miR-4721expression						
Low vs high	0.007	3.931	1.441–10.723	0.036	2.956	1.071–8.163
FOXA1 expression						
Low vs High	0.277	1.684	0.658–4.306			

## Discussion

Our previous research indicated that miR-4721 overexpressing was able to promote NPC cell growth by directly targeting GSK3β and subsequently mediated the PI3K/AKT/C-JUN/Sp1 pathway induced by EBV-miR-BART22 [14]. However, the stemness and invasion and metastasis of NPC cells fall outside the scope of the aforementioned study. In the current study, we further demonstrated the mechanism and function of miR-4721, which and showed that miR-4721 and FOXA1 reciprocally controlled their expression via a feedback loop and then activated Nanog and the downstream regulator of stem cell signaling to promote NPC stem cell enrichment and metastasis.

Similar to stem cells, tumor sphere are usually able to proliferate and self-renew, and are more tumorigenic [18, 19]. In the current study, we showed that miR-4721 overexpression resulted in increased SP cells, ALDH1 + cell rate, and tumor sphere formation. These results suggest that NPC tumor sphere cells are related to cancer stem-like populations. Tumor CSCs are often closely associated with EMT. Functionally, miR-4721 overexpression reinforced migration, and this effect can be reversed by the invasion of NPC cells and the addition of inhibitors to NPC cells overexpressing miR-4721. Further, miR-4721 overexpression exhibited a significant increase in metastasis in vivo. Our data verify that miR-4721 aggravates NPC stemness and metastasis.

The transcription factor FOXA1, an earlier cloned member of the Fox family closely related to the occurrence and development of some tumors [20]. In the current study, we found that FOXA1 was poorly expressed in NPC, which is consistent with the results obtained by Wang et al. [21]. Nanog is one of the core transcription factors that maintain the pluripotency and self-renewal of stem cells [22]. It is highly correlated with the occurrence of various tumors and participates in the maintenance of the characteristics of cancer stem cells [23–25]. Studies have shown that in human SCC cells, Nanog can directly bind to the promoters of ZEB1, ZEB2, Twist1, Prrx1, and miR-21; in human primary keratinocytes, Nanog can also induce EMT characteristics [26]. In ovarian cancer, Nanog can regulate E-Cadherin expression to regulate cell invasion and metastasis [27], whereas in colon cancer, it can induce snail and slug expression [28]. Luo et al. determined that Nanog as an unfavorable prognostic factor and was closely related to the invasive ability of NPC; moreover, Nanog and OCT4 were positively correlated in NPC, and E-Cadherin expression was negatively correlated in NPC [29]. Chen et al. reported on the endogenous binding of FOXA1 and Grg3 to the Nanog promoter − 2 kb region [17]. In the current study, we found that FOXA1 binds to the specific promoter TFBS of Nanog and inhibits its transcription. We further verified that miR-4721 overexpression could inhibit the expression of FOXA1 and E-Cadherin, as well as promote the expression of Nanog, ZEB2, and Snail. The aforementioned mechanism studies indicated that miR-4721 could directly target FOXA1 and promote the expression of Nanog to regulate downstream EMT-related markers and stem cell-related surface markers to promote NPC metastasis and stem cell enrichment, thereby participating in the pathogenesis of NPC.

Occupying the promoters of miR-4721, FOXA1 increased miR-4721 expression; such a loop might well lead to FOXA1 reactivation. Therefore, miR-4721 does not independently promote metastasis and stem cell enrichment; instead, it forms a negative feedback loop with FOXA1. The regulatory network formed by transcription factors and miRNA has been extensively studied. The feedback loop is composed of transcription factors and miRNA. It can be divided into a positive feedback loop and a negative feedback loop on the basis of the mode of action. Its mode of action is that transcription factors (TFs) and miRNA regulate each other [30]. Existing studies have found that feedback loops play an important role in various biological processes, particularly in maintaining homeostasis and regulating cell differentiation [31]. In our previous research, miR-3188 regulated NPC proliferation and chemosensitivity via a FOXO1-modulated positive feedback loop. In the current study, miR-4721 as a tumor promoter had a crucial role in the FOXA1 regulatory network and thus provided an insight into the FOXA1-mediated regulation of Nanog. Obviously, this complex regulatory network may be necessary for cells to maintain normal metastasis and stem cell enrichment. Interruption of this balance, such as miR-4721 overexpression, can lead to cell malignancy.

Our previous studies identified the association of high miR-4721 expression with poor prognosis in NPC. In this study, miR-4721 expression was negatively correlated with FOXA1 and FOXA1 was negatively correlated with Nanog expression in NPC tissues. NPC patients with low miR-4721 expression levels showed higher FOXA1 expression levels. Unlike miR-4721, the expression of FOXA1 was positively correlated with the overall survival of NPC patients. Patients exhibiting low miR-4721 expression had longer survival time than did those with high miR-4721 expression, regardless of their FOXA1 expression. These results suggest that miR-4721 can potentially regulate FOXA1 expression in human NPCs. Therefore, miR-4721 may be a potential target for the treatment of nasopharyngeal carcinoma.

**Fig. 7:**
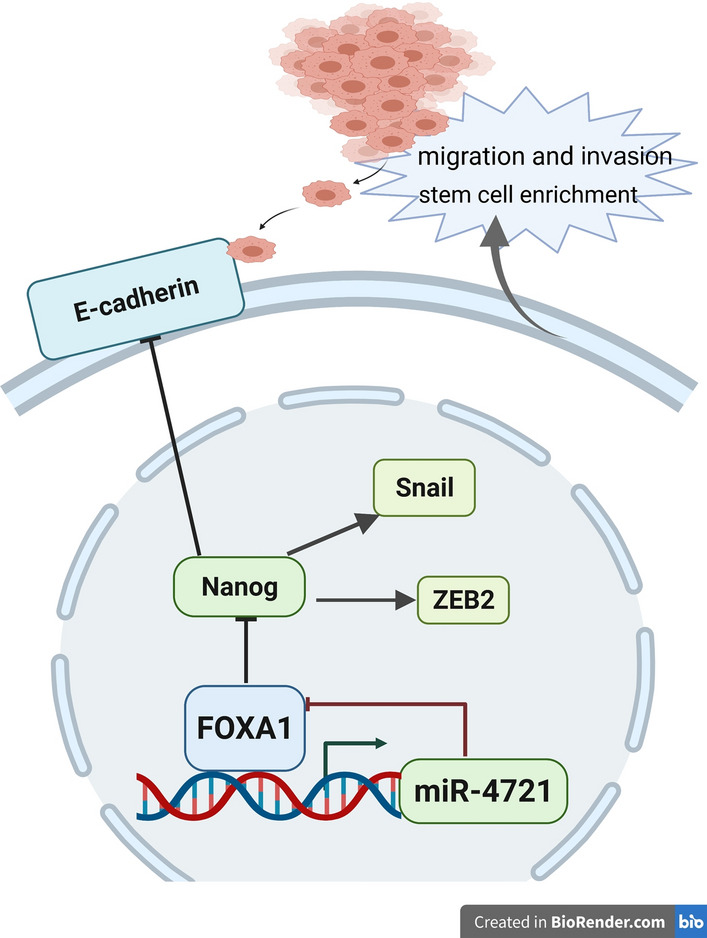
A schematic for miR-4721 forms a negative feedback loop with FOXA1. MiR-4721 could directly target FOXA1 to regulate downstream EMT-related markers and stem cell-related surface markers and FOXA1 in turn upregulates miR-4721 directly binding to its promoter

In summary, miR-4721 acts as a stemness accelerator in addition to its function as a tumor promoter via the miR-4721/FOXA1/Nanog negative feedback loop mechanism (Fig. 7). Although miRNA-based therapeutics have yet to be fully developed, miR-4721 exhibits potential.

## Supplementary Information

**Additional file 1: Table S1.** The sequences used in this study. **Table S2.** The primers used in this study. **Table S3.** A list of antibodies used for WB, ChIP, EMSA, IF and IHC. **Table S4.** The sequences used in Electrophoretic mobility shift assay.

## Data Availability

The data that support the findings of this study are available from the corresponding author upon reasonable request.

## Abbreviations

NPC: Nasopharyngeal cancer
miR: MicroRNA
NC: Negative control
EMT: Epithelial–mesenchymal transition
qRT-PCR: Quantitative real-time polymerase chain reaction
CSCs: Cancer stem cells
SP: Side population
CHIP: Chromatin immunoprecipitation
EMSA: Electrophoresis mobility shift assay
EBV: Epstein–Barr virus
ALDH1: Aldehyde dehydrogenase 1
