# Supplementary material for: A direct negative feedback loop of miR-4721/FOXA1/Nanog promotes nasopharyngeal cell stem cell enrichment and metastasis
Source: J Transl Med. 2021 Sep 9;19:387. doi: 10.1186/s12967-021-03059-y (PMC8428129; doi:10.1186/s12967-021-03059-y)
Supplement: Supplementary file 1 — Additional file 1: Table S1. The sequences used in this study. Table S2. The primers used in this study. Table S3. A list of antibodies used for WB, ChIP, EMSA, IF and IHC. Table S4. The sequences used in Electrophoretic mobility shift assay. [file 12967_2021_3059_MOESM1_ESM.docx]

Table S1.

The sequences used in this study.

| Gene | | | Sequence | Company |
| --- | --- | --- | --- | --- |
| miR-4721 mimics |  | Sense | 5’-UGAGGGCUCCAGGUGACGGUGG-3’ | RiboBio, Guangzhou, China |
|  |  | Antisense | 5’-ACUCCCGAGGUCCACUGCCACC3’ |  |
| Negative control |  | Sense | 5’ UUUGUACUACACAAAAGUACUG 3’ |  |
|  |  | Antisense | 5’ CUGUUCUUUUGUGUUGUUCUUU 3’ |  |
| miR-4721 inhibitor | | | 5’-ACUCCCGAGGUCCACUGCCACC3’ |  |
| Inhibitor negative control | | | 5’ CUGUUCUUUUGUGUUGUUCUUU 3’ |  |
| In situ hybridization probe sequence of miR-4721 | | | 5’ CCACCGTCACCTGGAGCCCTCA3’ | Axl-Bio, GuangZhou, China |

Table S2.

The primers used in this study.

| Primers name | | Sequence (5’-3’ ) | Company |
| --- | --- | --- | --- |
| FOXA1 | Forward | GCGCGAATTCAACCACCCGTTCTCCATCAA | Invitrogen, Shanghai, China |
|  | Reverse | GCGCCTCGAGTCATTGGTAGTACGCCGGCTCCAG |  |
| β-actin | Forward | TGACAAGGACAGGGTCTTCC |  |
|  | Reverse | CACCGTCCGTTGTATGTCTG |  |
| U6 | Forward | CTCGCTTCGGCAGCACA |  |
|  | Reverse | AACGCTTCACGAATTTGCGT |  |
| miR-4721 |  | UGAGGGCUCCAGGUGACGGUGG |  |
| Promoter of miR-4721-A | Forward | CAGAGGCTGTACCCTAAAGAAG | Axl-Bio, GuangZhou, China |
|  | Reverse | CTGAACCCTCCCACCTAAATAC |  |
| Promoter of miR-4721-B | Forward | AAAGGTGGGCCTGAGTTTC |  |
|  | Reverse | TGCAGTGCCTGTGATCATATT |  |
| Promoter of miR-4721-C | Forward | CTGAAGTCTGTGCAGAAGCTA |  |
|  | Reverse | TAGAGAGAGTGCGTGGGAA |  |
| Promoter of Nanog 1 | Forward | TCCGGAATGGTAGTCTGAGAA |  |
|  | Reverse | CTCCCACACAAGCTGACTTT |  |
| Promoter of Nanog 2 | Forward | CGCCCAATTTCATTTCCTTGTT |  |
|  | Reverse | ACTCTGCTCCTGGGTCTG |  |

Table S3.

A list of antibodies used for WB, ChIP, EMSA, IF and IHC.

| Antibodies name | Cat. No | Company | Species | Dulution |
| --- | --- | --- | --- | --- |
| FOXA1 | 53528 | CST | Rabbit | 1:400 (IHC); 1:1000 (WB); 1:100 (IF) |
| NANOG | 8822 | CST | Rabbit | 1:1000 (WB); 1:100 (IF) |
| SNAIL | 3879 | CST | Rabbit | 1:1000 (WB) |
| E-Cadherin | 3195 | CST | Rabbit | 1:1000 (WB) |
| ZEB1 | 3396 | CST | Rabbit | 1:1000 (WB) |
| ZEB2 | 97885 | CST | Rabbit | 1:1000 (WB) |
| β-actin | 60008-1-Ig | Proteintech | Mouse | 1:5000 (WB) |
| IgG | 2729 | CST | Rabbit | 1mg/ml(CHIP) |

Table S4.

The sequences used in Electrophoretic mobility shift assay.

| Gene | Type | | Sequence(5’-3’) | Company |
| --- | --- | --- | --- | --- |
| miR-4721 | probes | wild type | aacttggctgtttcctttccttctcctctcc | Axl-Bio, GuangZhou, China |
|  | competitors | wild type | aacttggctgtttcctttccttctcctctcc |  |
|  |  | mutant | aacttggcacaaaggaaagcttctcctctcc |  |
| Nanog | probes | wild type | ttttttgtttgtttgtttggttggt |  |
|  | competitors | wild type | accaaccaaacaaacaaacaaaaaa |  |
|  |  | mutant | ttttttgtccagccacctggttggt |  |
